# Coronavirus disease 2019 epidemic in impoverished area: Liangshan Yi autonomous prefecture as an example

**DOI:** 10.1186/s40249-020-00706-2

**Published:** 2020-08-12

**Authors:** Ru-Jun Liao, Chun-Nong Ji-Ke, Tao Zhang, Qiang Liao, Ling Li, Tian-Yu Zhu, Shao-Yong Bian

**Affiliations:** 1 Sichuan Center for Disease Control and Prevention, Chengdu, 610041 Sichuan Province China; 2 Liangshan Prefecture Center for Disease Control and Prevention, Xichang, 615000 Sichuan Province China; 3 Department of Epidemiology and Health Statistics, West China School of Public Health and West China Fourth Hospital, Sichuan University, Chengdu, 610041 Sichuan Province China

**Keywords:** SARS-CoV-2, Coronavirus disease 2019, Impoverished area, Epidemiology

## Abstract

**Background:**

The outbreak of coronavirus disease 2019 (COVID-19) had spread worldwide. Although the world has intensively focused on the epidemic center during this period of time, it is imperative to emphasize that more attention should also be paid to some impoverished areas in China since they are more vulnerable to disease outbreak due to their weak health service capacities. Therefore, this study took Liangshan Yi Autonomous Prefecture as an example to analyze the COVID-19 epidemic in the impoverished area, evaluate the control effect and explore future control strategies.

**Methods:**

In this study, we collected information including age, gender, nationality, occupation, and address of all COVID-19 cases reported from 25 January 2020 to 23 April 2020 in Liangshan Prefecture from the Nationwide Notifiable Infectious Diseases Reporting Information System (NIDRIS), which were used under license and not publicly available. Additionally, we retrieved other information of cases through epidemiological investigation reports reviewing. Data were analyzed using the software Excel 2010 and SPSS 17.0. The geographic distribution of cases was mapped using ArcGIS10.2.

**Results:**

By 23 April 2020, a total of 13 COVID-19 cases and two asymptomatic SARS-CoV-2 carriers were reported in Liangshan, in three family clusters. Among the cases, eight cases had a history of sojourning in Hubei Province (61.54%), of which six were related to Wuhan. Cases aged under 44 years accounted for 61.54%, with no child case. The delay of patients’ hospital visiting, and the low degree of cooperation in epidemiological investigation are problems.

**Conclusions:**

During the study period, Liangshan was well under control. This was mainly contributed to strict preventive strategies aimed at local culture, inter-sectoral coordination and highly degree of public cooperation. Besides, some possible environmentally and culturally preventive factors (e.g., rapid air flow and family concept) would affect disease prevention and control. In the next step, the health education about COVID-19 should be strengthened and carried out according to the special culture of ethnic minorities to enhance public awareness of timely medical treatment.

## Background

The novel coronavirus — 2019-nCoV, later named as severe acute respiratory syndrome coronavirus 2 (SARS-CoV-2), was identified on 7 January 2020 by Chinese scientists. Disease caused by this virus named COVID-19 by WHO. By 23 April 2020, COVID-19 has spread across over 160 countries and regions worldwide, infected 2 544 792 cases and caused 175 694 deaths [1]. The most common symptoms of COVID-19 are fever, tiredness, and dry cough. Some patients may have aches and pains, nasal congestion, runny nose, sore throat or diarrhea [2]. Older people, especially those with underlying medical problems like high blood pressure, heart problems or diabetes, are more likely to develop serious illness [2]. People with fever, cough and difficulty in breathing should seek medical attention (https://www.who.int/news-room/q-a-detail/q-a-coronaviruses).

Like SARS coronavirus (SARS-CoV) and Middle Eastern respiratory syndrome (MERS)-CoV, SARS-CoV-2 is a coronavirus that can be transmitted to humans, and these viruses are all related to high mortality in critically ill patients [3]. The COVID-19 now is a Category B infectious disease that has being managed as a Category A (most severe) infectious disease in China [4], requested network reporting in 2 h right after diagnosis.

Liangshan prefecture is the largest Yi community and one of the most impoverished areas in China, with backward infrastructure (except Xichang City) and weak public health condition, which has always been the main battlefield for the prevention and control of infectious diseases such as HIV. Because of the pleasant climate and high temperature in winter, Liangshan Prefecture attracts a large number of tourists during the Spring Festival holidays in China. In addition, there are a large number of labor imports and exports in Liangshan Prefecture, which brings the tide of returning home during the Spring Festival, and more challenges for the COVID-19 prevention and control. To date, several strategies have been implemented to reduce transmission and mitigate epidemic impact in Liangshan Prefecture. Three steps are necessary to take once an infectious disease outbreaks in certain regions, including controlling infectious sources, blocking the transmission routes, and protecting the susceptive population [5]. The confirmed cases and close contacts were quarantined to break the chain of infection. Site-specific disinfection was conducted to eliminate pathogens in the environment, so as to block the transmission by contact. People coming from high prevalence areas were tracked based on big data and were requested for daily health reporting. In response to this nationwide level one public health emergency, all scenic spots in Liangshan Prefecture were closed since 25 January 2020, and gradually re-opened from 24 February 2020. All public places of entertainment and most of the restaurants were closed since 25 January 2020. Based on mobile operator data, during 20 January–13 February 2020, the number of immigrants fell by 62% compared with the same period last year. The airport, railway station, expressway entrance and exit have been set up inspection checkpoints. Centralized quarantine places have been set up in each county and city for close contacts, as well as for persons returning from areas affected by the outbreak. Dinner parties are banned, catering packaging and takeout are encouraged, and entertainment venues and factories where people gather are closed. Residents are advised to take personal precautions and stay indoors. Judging from the number of confirmed cases, the epidemic situation in Liangshan Prefecture is not serious.

This study analyzed the epidemiological characteristics of COVID-19 cases and family clusters in Liangshan Prefecture, evaluated the prevention and control effect on COVID-19, and proposed the next step of prevention and control measures. Meanwhile, in-depth analysis of some epidemiological characteristics was conducted to explore the possible reasons.

## Methods

### Study area

Liangshan Prefecture is a Yi autonomous prefecture in Sichuan Province, China, including both majority (Han) and minority (Yi, Tibetan) areas. Liangshan Prefecture is located in latitude 26°03′–29°18′N and longitude 100°03′–103°52′E, measures 60 400 km^2^ and consists of 1 city and 16 counties. The city is named as Xichang, of which residents are mainly Han people. The counties are named as follows: (1) Yanyuan, (2) Dechang; (3) Huili; (4) Huidong; (5) Ningnan; (6) Puge; (7) Butuo; (8) Jinyang; (9) Zhaojue; (10) Xide; (11) Mianning; (12) Yuexi; (13) Ganluo; (14) Meigu; (15) Leibo; (16) Muli. Among them, residents in Yanyuan, Puge, Butuo, Jinyang, Zhaojue, Xide, Yuexi, Ganluo, Meigu, Leibo counties are mainly Yi minority people. Residents in Dechang, Huili, Huidong, Ningnan, Mianning counties are mainly Han people. And Muli is a Tibetan autonomous county. Those 11 minority counties also defined as state-level poverty-stricken counties.

### Data sources

All cases of COVID-19 reported from 25 January to 23 April 2020 in Liangshan Prefecture were involved in this study. And information including the patient’s age, sex, nationality, occupation, and address were extracted from the Nationwide Notifiable Infectious Diseases Reporting Information System (NIDRIS) which were used under license and not publicly available. Additionally, we retrieved other case information by epidemiological investigation reports review. Maps of Liangshan Prefecture were downloaded from Data Sharing Infrastructure of Earth System Science (http://www.geodata.cn/).

### COVID-19 case definition

The diagnosis of COVID-19 is based on the Diagnosis and Treatment Scheme of Pneumonia Caused by Novel Coronavirus established by National Health Commission of the People’s Republic of China [6–8]. The clinical manifestations of COVID-19 mainly includes fever, fatigue and dry cough. A few patients also combine with nasal congestion, runny nose, sore throat, diarrhea and other symptoms. Dyspnea and/or hyoxemia usually occur in severe cases a week later. Severe cases rapidly progress to acute respiratory distress syndrome, irreformable metabolic acidosis, bleeding and coagulation dysfunction and other fatal symptoms. Mild cases present low fever, mild fatigue, with no pneumonia. Cases with symptoms are confirmed after testing positive for viral nucleic acid, while those without any symptoms but test positive were defined as asymptomatic carriers.

### Statistical analysis

Data were analyzed using the software Excel 2010 (Microsoft Corporation, Redmond, USA) and SPSS 17.0 (International Business Machines Corporation, Armonk, New York, USA). Since the sample size was small and there was a maximum value, data of the age, time of last exposure to onset, time of onset to see a doctor, time since visit hospital to diagnose, time of hospital stay were described using median (interquartile range [IQR]). The geographic distribution of cases was mapped using ArcGIS10.2 (Environmental Systems Research Institute, Redlands, California, USA).

## Results

### Demographic characteristics

By 23 April 2020, a total of 13 diagnosed COVID-19 cases were reported in Liangshan Prefecture during the epidemic, who were all included in the analysis. Since most of the cases were imported, the incidence rate was not calculated. The male to female sex ratio of cases was 1.6:1. The patients were aged from 23 to 66 years, with a median age of 37 (IQR: 28.5, 59.5) years. Cases aged under 44 years accounted for 61.54%. There were ten cases of Han nationality and three cases of Yi nationality. Occupation of cases mainly included commercial service (four cases), farmers (three cases) and retired personnel (three cases) (Table 1).

**Table 1:** All COVID-19 cases in Liangshan Prefecture

No.	Age	Gender	Nationality	Location	The earliest suspected exposure	The last suspected exposure	Onset date	Date of hospital visiting	Diagnosis date	Date of discharge
1	28	Female	Han	Yanyuan	15 Jan	18 Jan	19 Jan	23 Jan	24 Jan	9 Feb
2	32	Male	Han	Yanyuan	15 Jan	19 Jan	24 Jan	24 Jan	25 Jan	14 Feb
3	43	Male	Yi	Ganluo	20 Jan	20 Jan	25 Jan	25 Jan	29 Jan	28 Feb
4	55	Male	Han	Xichang	19 Jan	19 Jan	24 Jan	30 Jan	31 Jan	15 Feb
5	23	Female	Yi	Ganluo	22 Jan	22 Jan	27 Jan	29 Jan	31 Jan	13 Feb
6	29	Female	Han	Xichang	17 Jan	20 Jan	24 Jan	25 Jan	2 Feb	13 Feb
7	64	Female	Han	Xichang	22 Jan	23 Jan	1 Feb	3 Feb	4 Feb	20 Feb
8	23	Male	Han	Xichang	22 Jan	22 Jan	28 Jan	2 Feb	6 Feb	22 Feb
9	53	Male	Yi	Ganluo	22 Jan	29 Jan	2 Feb	5 Feb	7 Feb	6 Mar
10	37	Male	Han	Xichang	25 Jan	3 Feb	6 Feb	8 Feb	8 Feb	22 Feb
11	32	Male	Han	Xichang	17 Jan	17 Jan	21 Jan	12 Feb	13 Feb	25 Feb
12	66	Male	Han	Xichang	25 Jan	3 Feb	15 Feb	14 Feb	15 Feb	2 Mar
13	64	Female	Han	Xichang	25 Jan	Feb 3	15 Feb	14 Feb	15 Feb	2 Mar

### Temporal pattern

The time sequence of COVID-19 cases has no obvious pattern in Liangshan Prefecture through graphical observation. The first case was reported on 24 January 2020, nine cases were sporadic imported cases. The last case was reported on 15 February, while no case was reported in the later 14 days.

The median time from first exposure to onset was 7 days (IQR: 5, 11), ranged from 3 to 20 days), from last exposure to onset was 5 days (IQR: 3.5, 7.5), ranged from 1 to 12 days, while the median time from onset to see a doctor was 2 days (IQR: 0, 4.5), ranged from 0 to 22 days. The median time since visit hospital to diagnose was 1 days (IQR: 1, 3), ranged from 0 to 8 days. Twelve patients in 13 were discharged by 2 March 2020. The median time of hospital stay was 15 days (IQR: 13.5, 18.0), ranged from 11 to 30 days. In addition, the time of hospital stay of case No. 3 was particularly long mainly due to head trauma by accident.

### Spatial pattern

**Fig. 1:**
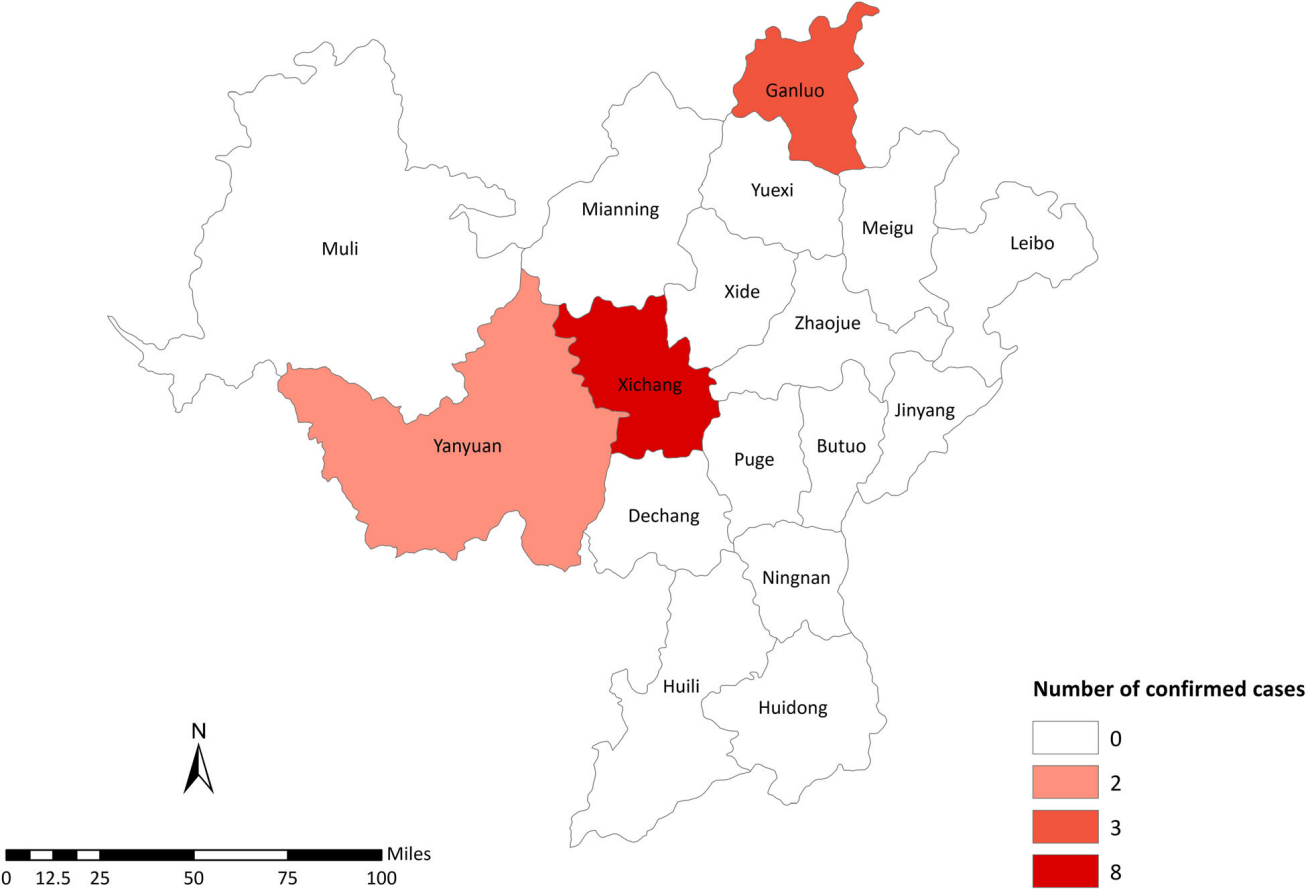
Distribution of COVID-19 cases in Liangshan Prefecture

Among the reported cases, eight cases were from Xichang City, three cases from Ganluo County, and two cases from Yanyuan County. Among the 13 confirmed cases, 8 had a history of sojourning in Hubei province (61.54%), of which 6 were related to Wuhan. (Fig. 1).

### Spread spectrum

**Fig. 2:**
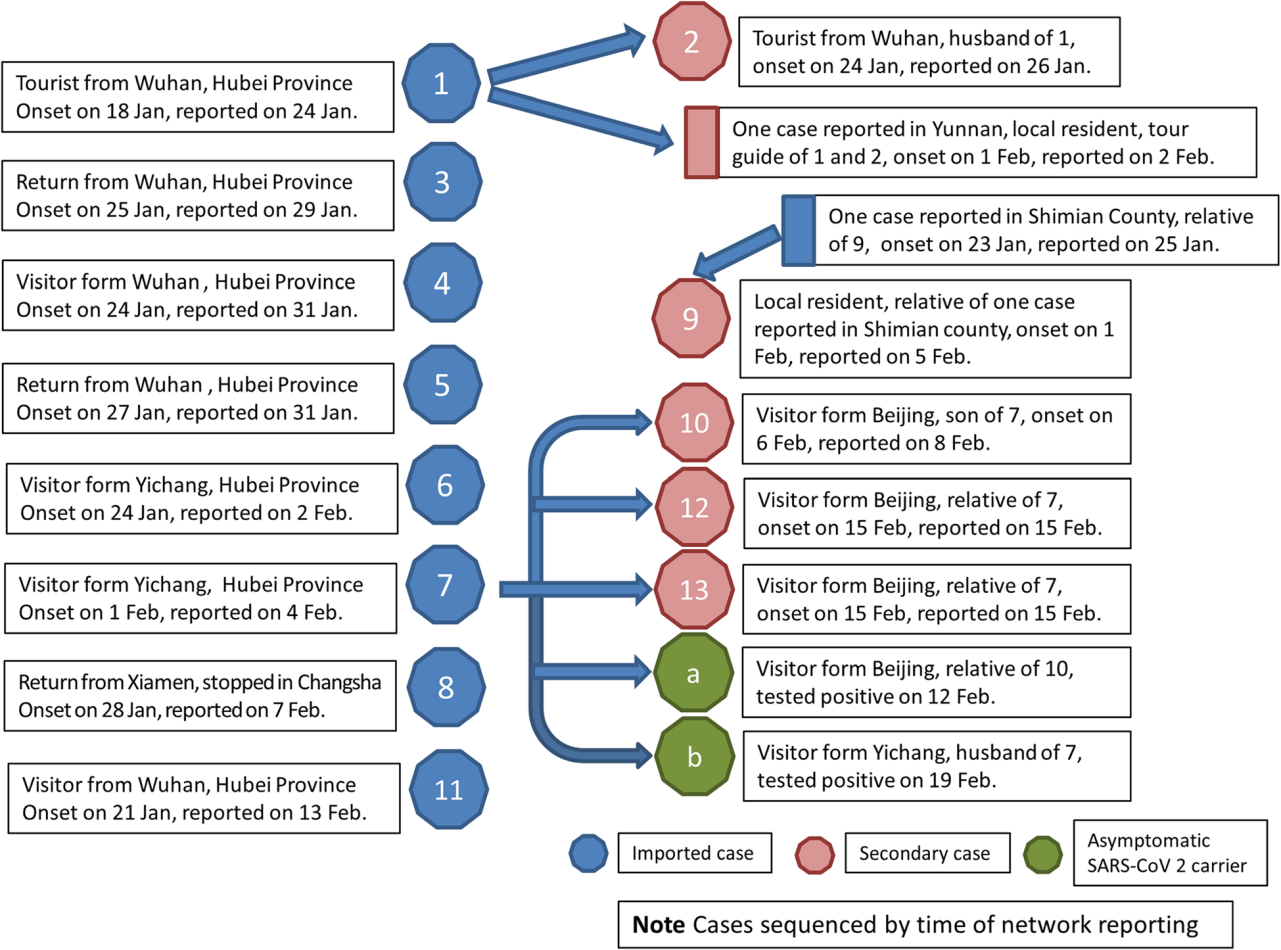
Spread Spectrum of COVID-19 in Liangshan Prefecture

Based on the epidemiological survey, we mapped the spread spectrum of all cases in Liangshan Prefecture. Except those nine imported cases, all 4 second-generation cases traced back to the source of infection (Fig. 2), with no third-generation case. The second-generation cases mainly onset in intensive quarantine sites, except case No.9. The main reason that case No.9 was not included in the management as close contact was that the patient concealed the contact history because of the misunderstanding of management policy. No case developed severe symptoms such as acute respiratory distress syndrome (ARDS). There was no significant difference in symptom severity between cases of first generation and second generation.

### Family clusters

As shown in the spread spectrum of COVID-19 in Liangshan Prefecture, there were three family clusters. One couple of tourists from Wuhan to Lugu Lake scenic area were involved in the first incident, and infected one of their tour guides. As part of the Lugu Lake scenic area belongs to Yanyuan County of Liangshan Prefecture, part belongs to Ninglang County of Lijiang (Yunnan Province), the tour guide accepted quarantine in Lijiang, then he was reported onset there. The second cluster was due to family feasts. The third cluster of cases occurred among family members who lived together, and, of concern, a 6-year-old girl who had not been infected.

### Close contact tracing

Based on 13 confirmed COVID-19 cases, a total of 627 close contacts were traced and quarantined. Except for four cases onset in quarantine place, the rest of each case produced an average of 70 close contacts.

### Asymptomatic SARS-CoV 2 carriers

By 26 April 2020, two asymptomatic SARS-CoV-2 carriers were detected and reported in Liangshan Prefecture, and both of them were close contacts in quarantine place. They were tested positive on 12 Feburary and 19 February respectively, and released quarantine on 12 April after twice negative result of nucleic acid tests.

### Arrivals from abroad

Because of the rapid increase in COVID-19 cases worldwide, recent arrivals from out of China were requested for 14 days’ quarantine and nucleid acid detection. By 23 April 2020, 364 arrivals were tested in Liangshan Prefecture, and the results were all negative.

### Effect of prevetion and control

During the study period, the epidemic of COVID-19 in Liangshan was well under control. This was mainly contributed to strict preventive strategies, iner-sectoal coordination and highly degree of public cooperation (Table 2).

**Table 2:** Milestones of COVID-19 prevention and control in Liangshan Prefecture

Date	Milestones
19 January	Identified and publicized designated treatment hospitals for COVID-19.
23 January	The leading group of COVID-19 joint prevention and control in Liangshan Prefecture was established
24 January	Level one emergency response activated in Sichuan.
25 January	a. All the scenic spots, public places of entertainment, most restaurants in Liangshan Prefecture were closed. b. Tracking of Wuhan returnees started. All traced Wuhan returnees were required for 14 days of home quarantine with daily health report. c. Residents were advised to wear masks and stay indoors.
26 January	a. Investigations were expanded to visitors from severely affected areas. b. Mobile messages, WeChat public accounts, websites and TV were used to publicize the COVID-19 related knowledge and prevention strategies.
27 January	a. Use the big data of public security and provincial government to strengthen the screening and management of contacts and people returning from epidemic-related areas. b. All the county-level CDCs started to provide public advisory services on COVID-19 prevention and control on hotlines.
2 February	Returnees from outside Liangshan Prefecture were requested for 14 days of home quarantine.
17 February	Those who go out of Liangshan Prefecture and return were required to obtain health certificates.
24 February	The scenic area was gradually opened up under strict registration in entrance and flow limitation.
26 February	The emergency response level of epidemic prevention and control in Sichuan Province has been adjusted from the first level to the second level.
27 February	All the 17 counties were classified into middle risk areas and low risk areas to conduct differentiated prevention and control measures.
25 March	The emergency response level of epidemic prevention and control in Sichuan Province had been adjusted from the second level to the third level. The control measures of the passageway from Hubei Province except Wuhan were lifted.
26 March	Quarantine and nucleic acid testing were requested to carry out for all recent arrivals from Hubei Province and abroad.
1 April	Senior high students returned to school.
7 April	Grade three junior high students returned to school.
8 April	The control measures of the passageway from Wuhan were lifted.
15 April	Middle schools were open for all grades.
20 April	Secondary schools were open for grade four, five and six.
27 April	Secondary schools were open for all grades.

## Discussion

Over the past few months, COVID-19 has spread across over 160 countries and regions worldwide from six continents [1]. Mortality rate went up rapidly within a short period of time and threatened lives of the entire global population. The Chinese people have made unprecedented efforts to combat the epidemic. In the outbreak response, the Chinese government has quickly taken actions to contain its spread inside China, including early detection, early diagnosis and reporting, early isolation and treatment, tracing all possible contacts to be under quarantine, promoting basic hygiene measures to the public such as frequent hand washing, cancelling public gathering, closing schools, extending the Spring Festival holiday, delaying return to work, and, as the most severe measure, outbound travel restrictions of the city of Wuhan [9, 10].

With the largest concentration of Yi nationality (> 50%) and as one of the most impoverished areas in China, Liangshan Prefecture represents some unique cultural characteristics, backward infrastructure conditions (except Xichang City) and weak public health landscape, which put forward more challenges for the COVID-19 prevention and control.

### Epidemiological characteristics of COVID-19 in Liangshan prefecture

The cases of COVID-19 in Liangshan Prefecture were sporadic, mainly located in Xichang City, Yanyuan County and Ganluo County., while no cases have been reported in other counties. The confirmed cases were mainly imported from Hubei province. The second-generation cases mainly onset in the intensive quarantine sites, with no third-generation case and no community transmission.

Over 60% of patients infected by SARS-CoV-2 were male, which was similar to earlier studies [2, 11]. The phenotype of more male cases than female cases in Liangshan Prefecture may be because that men were more frequently involved in social activities such as dinner parties.

The majority of cases were in young people, with a median age of 37 years. The patients were younger in our study than in previous studies [2, 11]. Cases aged under 44 years accounted for 61.54% (8/13). There was no child onset in Liangshan Prefecture, which might be due to parents’ intensive protection measures for children. It is also possible that children may have been infected invisibly, and the current testing techniques are not yet able to determine their status. We have stored blood samples from all close contacts and will test them again when the antibody testing technology is more accurate.

There were three family clusters of COVID-19 in Liangshan Prefecture. The case No.1 and No.7 as sources of infection of clusters were highly infective. However, because of the timely tracing and quarantine of close contacts, all second generation of cases were onset in quarantine place, with no three generation of cases emerged. Therefore, the key to control the spread of COVID-2019 is timely tracing and management of close contacts. In most scenarios, highly effective contact tracing and case isolation is enough to control a new outbreak of COVID-19 within 3 months [12].

### New findings

We found that although some cases took a long time from onset to isolation in Xichang City, except for second-generation cases within the family, no transmission has occurred outside, which may be related to the rapid air flow. Due to geographical reasons, the wind in Xichang is very strong, especially in winter and spring. Previous studies by scholars have found that air supplied horizontally to the breathing zone intensified the mixing between the exhaled air and the room air, which reduced the exposure risk of persons in close proximity and flattened the risk-distance curves [13].

In our study, fever is the most common symptom in patients with COVID-19, which is in accordance with previous studies, but not all patients had a fever [2, 11, 14]. The delay of fever manifestation hinders early identification of patients infected with SARS-CoV-2, especially for asymptomatic patients that is more difficult for the identification of suspected cases [15].

We also found that it is very important that virus nucleic acid detection should be conducted on all the close contacts at least twice before they are relieved quarantine. The twice detections was important to exclude asymptomatic infections. In the third cluster, four close contacts were tested positive during quarantine, one of them was tested negative on the last day of quarantine. After 14 days of quarantine, the close contacts in Liangshan Prefecture were requested 14 days’ home quarantine, and virus nucleic acid detection on the 7th day and 14th day of home quarantine.

Except four cases onset in quarantine place, each case produced an average of 70 close contacts, which indicated that health education for the public was not good enough at the beginning of the COVID-19 outbreak.

The family branch has a very important position and function in the society of the Yi nationality. Internally, the family branch maintains social order and protects its members’ safety, handles various affairs, unites, helps each other, rescues and educates its members, and externally, in the name of the family branch, conquers the enemy or makes alliances. Therefore, the core of Yi culture lies in protection. Furthermore, the low literacy might lead to misunderstanding of prevention and control measures, resulting in the reservation of close contacts’ information. When there is a COVID-19 case in the family, members of the whole family branch may deny having no contact with the case because they have a poor understanding of the disease and/or a misunderstanding of the quarantine. As a result, some close contacts were not quarantined in time. The probability of control decreases with long delays from symptom onset to isolation, fewer cases ascertained by contact tracing, and increasing transmission before symptoms [8]. Fortunately, there was no third-generation case because of quarantine. Based on the experience of case No.9, we suggested that an investigation team was composed of a disease control worker, a township cadre and a public security officer for Yi patients in rural areas.

Fragile economies, scarce health resources and poor health care practices often give rise to the high incidence of disease in impoverished areas. However, according to this retrospective study of the COVID-19 epidemic in Liangshan Prefecture, no additional severe epidemic has been found there, which is better than other areas. This may be a result of government regarding public health as the highest priority, the unprecedentedly stringent prevention and control measures, and the tremendous amount of assistance from various medical and health institutions to the local area. However, this study still found that low literacy and some other factors are important obstacles to the effective implementation of prevention and control measures. Therefore, the results show that although the implement of centralized and assault prevention and control measures in impoverished areas can play a certain role, long-term mechanism construction of disease prevention and control in those areas should not be neglected. Only by ensuring long-term and sustained medical resource input and effective health education in impoverished areas can we truly improve the overall response and treatment capacity in those areas to new and re-emerging infectious diseases, so as to minimize the risk of the epidemic to the people in those areas.

### Proposition for prevention and control work in the next step

Firstly, the next step of prevention and control work can be based on the big data from the public security and the three major mobile operators to track the import personnel from high-risk areas. Secondly, the management of cough and fever medicine in the pharmacy should be strengthened. Thirdly, the purchase records should be kept and feedback to the community health agencies, so as to provide clues for the early detection of cases. Fourthly, we should strengthen the monitoring and tracing of the patients in the fever clinics. Since the asymptomatic carriers can also be the source of infection [15], the monitoring on the asymptomatic carriers and cured cases is necessary. At the same time, antibody IgG and IgM testing should be carried out in healthy people to detect the level of herd-immunity level. Importantly, it is still suggested that people wear masks when they go out, and avoid unnecessary gathering. To sustain current gains, we must be vigilant.

There are still some limitation of this study: (I) Since there were only 13 cases in this study, most of which were imported, it was impossible to calculate the incidence rate or analyze the trend of epidemic. (II) Because of the difficulty of sharing information across regions, we cannot trace the source of infection of the eight imported cases. (III) Due to the problem of test positive in cured cases, the discharge standard of cases is still controversial at present. The discharge standard adopted in Liangshan Prefecture is two times of nucleic acid test negative after the complete disappearance of symptoms, which may still be inaccurate. (VI) The COVID-19 pandemic is still on, although there was no new case reported after 15 February, we cannot conclude that the spread of the disease has ceased in Liangshan Prefecture. (V) As the serum antibody detection technology is inaccurate by now, we have not yet been able to detect latent infections in the population.

## Conclusions

Under strict preventive strategies aimed at the local culture, with inter-sectoral coordination and high degree of public cooperation, the current outbreak of COVID-19 in Liangshan Prefecture is well controlled. However, there are still shortcomings in the work of prevention and control needing to be improved. The cultural characteristics and low educational level of residents in some areas may be the main reasons for the delay of hospital visiting and the low degree of cooperation in epidemiological investigation. Therefore, COVID-19 related health education should be strengthened and carried out according to the special culture of ethnic minorities to enhance public awareness of timely medical treatment, and multi-sector cooperation needs to be enhanced. By making better use of big data, we can fully and timely grasp the activity track of cases before onset, to track and isolate close contacts as soon as possible.

Nevertheless, the prevention and control measures such as closing scenic spots, industries shutting down also resulted in great loss to the economy of Liangshan Prefecture. The next step of prevention and control work is mainly to prevent imports, detect the asymptomatic SARS-CoV-2 carriers, and gradually and orderly resume normal production, get normal social and economic order returned. Moreover, the scientific communities, governments and non-governmental organizations in different fields, such as public health, agriculture, ecology, epidemiology, governance planning, science need to collaboratively prevent future outbreaks, with better coordination.

## Data Availability

No data and materials are available for sharing.

## Abbreviations

SARS-CoV-2: Severe acute respiratory syndrome associated coronavirus 2
COVID-19: Corona virus disease 2019
CDC: Center for Disease Control and Prevention
HIV: Human immunodeficiency virus
WHO: World Health Organization

## References

[1] World Health Organization. Coronavirus disease 2019 (COVID-19): situation report, 94: World Health Organization; 2020. https://apps.who.int/iris/handle/10665/331865.

[2] Chen N, Zhou M, Dong X, Qu J, Gong F, Han Y (2020). Epidemiological and clinical characteristics of 99 cases of 2019 novel coronavirus pneumonia in Wuhan, China: a descriptive study. Lancet..

[3] Parry J (2020). Wuhan: Britons to be evacuated as scientists estimate 44 000 cases of 2019-nCOV in the city. BMJ..

[4] National Health Committee. Announcement of the National Health Committee of the People’s Republic of China; http://www.nhc.gov.cn/jkj/s7916/202001/44a3b8245e8049d2837a4f27529cd386.shtml. Accessed 15 Mar 2020.

[5] TNCPERE (2020). The epidemiological characteristics of an outbreak of 2019 novel coronavirus diseases (COVID-19) — China, 2020. China CDC Weekly.

[6] National Health Commission of People’s Republic of China (2020). Diagnosis and treatment of novel coronavirus pneumonia (trial version 3).

[7] National Health Commission of People’s Republic of China (2020). Diagnosis and treatment of novel coronavirus pneumonia (trial version 4).

[8] National Health Commission of People’s Republic of China (2020). Diagnosis and treatment of novel coronavirus pneumonia (trial version 5).

[9] General Administration of Quality Supervision (2016). Inspection and Quarantine of the People’s Republic of China, Standardization Administration of the People’s Republic of China.

[10] Wilder-Smith A, Freedman DO (2020). Isolation, quarantine, social distancing and community containment: pivotal role for old-style public health measures in the novel coronavirus (2019-nCoV) outbreak. J Travel Med.

[11] Huang C, Wang Y, Li X, Ren L, Zhao J, Hu Y (2020). Clinical features of patients infected with 2019 novel coronavirus in Wuhan, China. Lancet.

[12] Hellewell J, Abbott S, Gimma A, Bosse NI, Jarvis CI, Russell TW (2020). Feasibility of controlling COVID-19 outbreaks by isolation of cases and contacts. Lancet Glob Health.

[13] Ai ZT, Huang T, Melikov AK (2019). Airborne transmission of exhaled droplet nuclei between occupants in a room with horizontal air distribution. Build Environ.

[14] Wang D, Hu B, Hu C (2020). Clinical characteristics of 138 hospitalized patients with 2019 novel coronavirus-infected pneumonia in Wuhan, China. JAMA.

[15] Rothe C, Schunk M, Sothmann P, Bretzel G, Froeschl G, Wallrauch C (2020). Transmission of 2019-nCoV infection from an asymptomatic contact in Germany. N Engl J Med.

